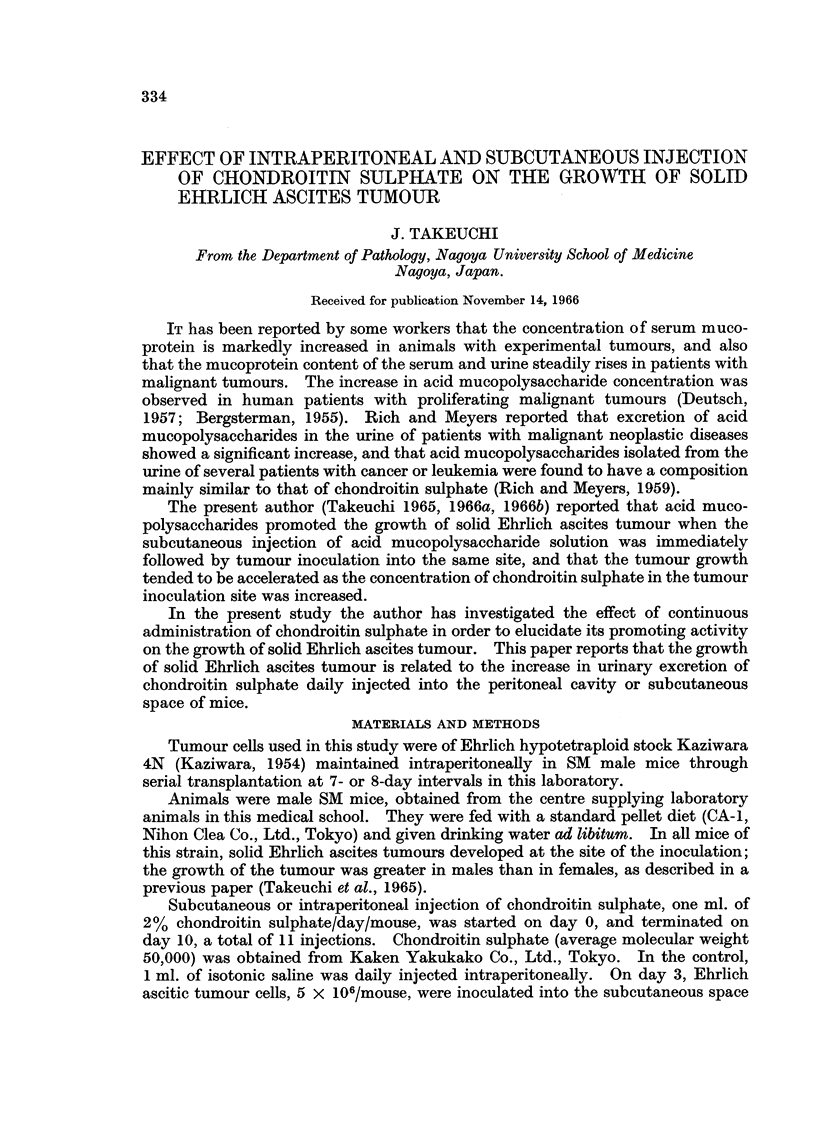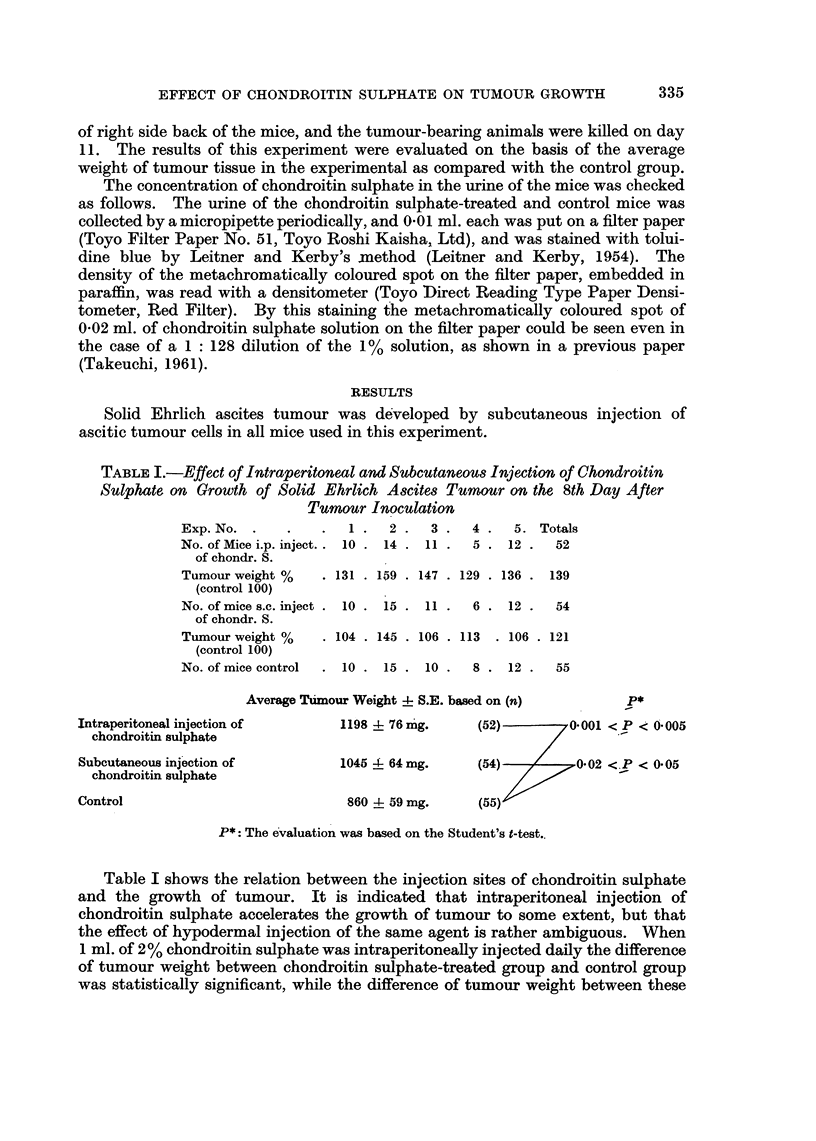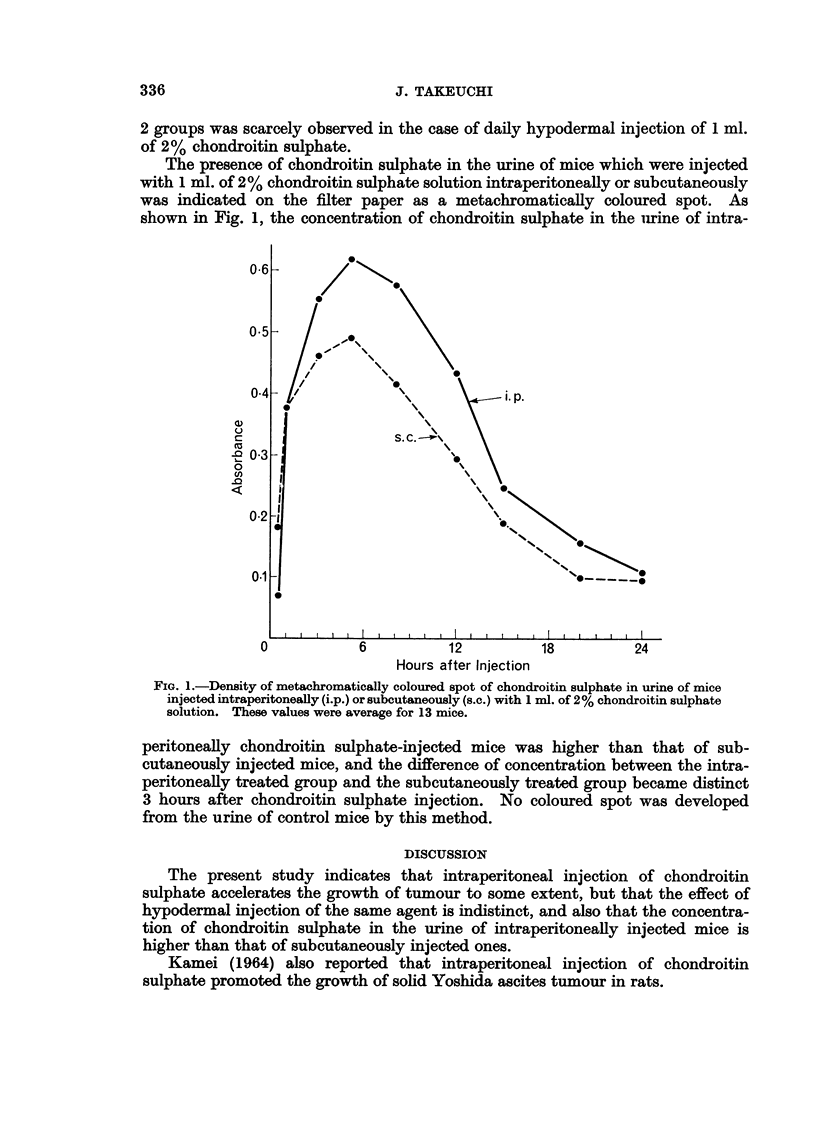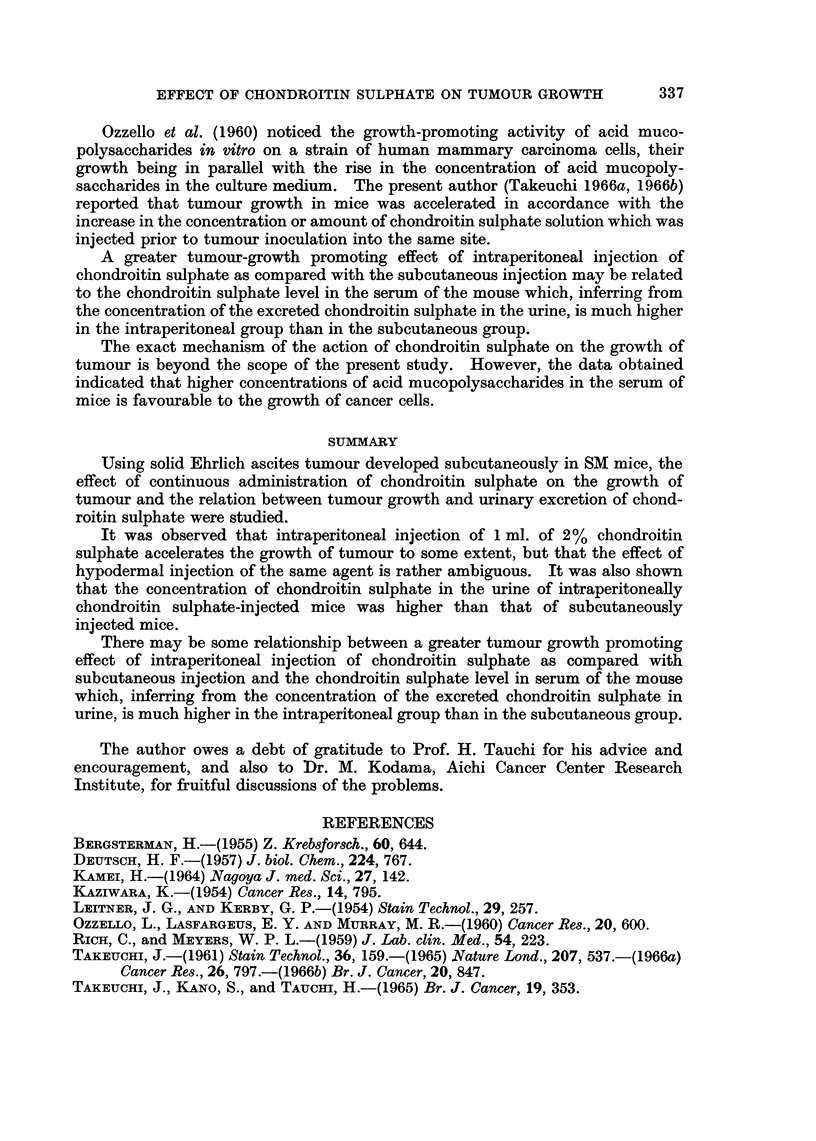# Effect of intraperitoneal and subcutaneous injection of chondroitin sulphate on the growth of solid Ehrlich ascites tumour.

**DOI:** 10.1038/bjc.1967.37

**Published:** 1967-06

**Authors:** J. Takeuchi


### EFFECT OF INTRAPERITONEAL AND SUBCUTANEOUS INJECTION OF CHONDROITIN SULPHATE ON THE GROWTH OF SOLID EHRLICH ASCITES TUMOUR

J. TAKEUCHI

*From the Department of Pathology, Nagoya University School of Medicine Nagoya, Japan.*

Received for publication November 14, 1966

It has been reported by some workers that the concentration of serum mucoprotein is markedly increased in animals with experimental tumours, and also that the mucoprotein content of the serum and urine steadily rises in patients with malignant tumours. The increase in acid mucopolysaccharide concentration was observed in human patients with proliferating malignant tumours (Deutsch, 1957; Bergsterman, 1955). Rich and Meyers reported that excretion of acid mucopolysaccharides in the urine of patients with malignant neoplastic diseases showed a significant increase, and that acid mucopolysaccharides isolated from the urine of several patients with cancer or leukemia were found to have a composition mainly similar to that of chondroitin sulphate (Rich and Meyers, 1959).

The present author (Takeuchi 1965, 1966*a*, 1966*b*) reported that acid mucopolysaccharides promoted the growth of solid Ehrlich ascites tumour when the subcutaneous injection of acid mucopolysaccharide solution was immediately followed by tumour inoculation into the same site, and that the tumour growth tended to be accelerated as the concentration of chondroitin sulphate in the tumour inoculation site was increased.

In the present study the author has investigated the effect of continuous administration of chondroitin sulphate in order to elucidate its promoting activity on the growth of solid Ehrlich ascites tumour. This paper reports that the growth of solid Ehrlich ascites tumour is related to the increase in urinary excretion of chondroitin sulphate daily injected into the peritoneal cavity or subcutaneous space of mice.

### MATERIALS AND METHODS

Tumour cells used in this study were of Ehrlich hypotetraploid stock Kaziwara 4N (Kaziwara, 1954) maintained intraperitoneally in SM male mice through serial transplantation at 7- or 8-day intervals in this laboratory.

Animals were male SM mice, obtained from the centre supplying laboratory animals in this medical school. They were fed with a standard pellet diet (CA-1, Nihon Clea Co., Ltd., Tokyo) and given drinking water *ad libitum*. In all mice of this strain, solid Ehrlich ascites tumours developed at the site of the inoculation; the growth of the tumour was greater in males than in females, as described in a previous paper (Takeuchi *et al.*, 1965).

Subcutaneous or intraperitoneal injection of chondroitin sulphate, one ml. of 2% chondroitin sulphate/day/mouse, was started on day 0, and terminated on day 10, a total of 11 injections. Chondroitin sulphate (average molecular weight 50,000) was obtained from Kaken Yakukako Co., Ltd., Tokyo. In the control, 1 ml. of isotonic saline was daily injected intraperitoneally. On day 3, Ehrlich ascitic tumour cells, $5 \times 10^6$/mouse, were inoculated into the subcutaneous space of right side back of the mice, and the tumour-bearing animals were killed on day 11. The results of this experiment were evaluated on the basis of the average weight of tumour tissue in the experimental as compared with the control group.

The concentration of chondroitin sulphate in the urine of the mice was checked as follows. The urine of the chondroitin sulphate-treated and control mice was collected by a micropipette periodically, and 0·01 ml. each was put on a filter paper (Toyo Filter Paper No. 51, Toyo Roshi Kaisha, Ltd), and was stained with toluidine blue by Leitner and Kerby's method (Leitner and Kerby, 1954). The density of the metachromatically coloured spot on the filter paper, embedded in paraffin, was read with a densitometer (Toyo Direct Reading Type Paper Densitometer, Red Filter). By this staining the metachromatically coloured spot of 0·02 ml. of chondroitin sulphate solution on the filter paper could be seen even in the case of a 1 : 128 dilution of the 1% solution, as shown in a previous paper (Takeuchi, 1961).

### RESULTS

Solid Ehrlich ascites tumour was developed by subcutaneous injection of ascitic tumour cells in all mice used in this experiment.

TABLE I.—*Effect of Intraperitoneal and Subcutaneous Injection of Chondroitin Sulphate on Growth of Solid Ehrlich Ascites Tumour on the 8th Day After Tumour Inoculation*

| Exp. No. | 1 | 2 | 3 | 4 | 5 | Totals |
|---|---|---|---|---|---|---|
| No. of Mice i.p. inject. of chondr. S. | 10 | 14 | 11 | 5 | 12 | 52 |
| Tumour weight % (control 100) | 131 | 159 | 147 | 129 | 136 | 139 |
| No. of mice s.c. inject of chondr. S. | 10 | 15 | 11 | 6 | 12 | 54 |
| Tumour weight % (control 100) | 104 | 145 | 106 | 113 | 106 | 121 |
| No. of mice control | 10 | 15 | 10 | 8 | 12 | 55 |

| | Average Tumour Weight ± S.E. | based on (*n*) | *P** |
|---|---|---|---|
| Intraperitoneal injection of chondroitin sulphate | 1198 ± 76 mg. | (52) | 0·001 < *P* < 0·005 (vs. control) |
| Subcutaneous injection of chondroitin sulphate | 1045 ± 64 mg. | (54) | 0·02 < *P* < 0·05 (vs. control) |
| Control | 860 ± 59 mg. | (55) | |

*P**: The evaluation was based on the Student's *t*-test.

Table I shows the relation between the injection sites of chondroitin sulphate and the growth of tumour. It is indicated that intraperitoneal injection of chondroitin sulphate accelerates the growth of tumour to some extent, but that the effect of hypodermal injection of the same agent is rather ambiguous. When 1 ml. of 2% chondroitin sulphate was intraperitoneally injected daily the difference of tumour weight between chondroitin sulphate-treated group and control group was statistically significant, while the difference of tumour weight between these

2 groups was scarcely observed in the case of daily hypodermal injection of 1 ml. of 2% chondroitin sulphate.

The presence of chondroitin sulphate in the urine of mice which were injected with 1 ml. of 2% chondroitin sulphate solution intraperitoneally or subcutaneously was indicated on the filter paper as a metachromatically coloured spot. As shown in Fig. 1, the concentration of chondroitin sulphate in the urine of intra-

FIG. 1.—Density of metachromatically coloured spot of chondroitin sulphate in urine of mice injected intraperitoneally (i.p.) or subcutaneously (s.c.) with 1 ml. of 2% chondroitin sulphate solution. These values were average for 13 mice.

peritoneally chondroitin sulphate-injected mice was higher than that of subcutaneously injected mice, and the difference of concentration between the intraperitoneally treated group and the subcutaneously treated group became distinct 3 hours after chondroitin sulphate injection. No coloured spot was developed from the urine of control mice by this method.

### DISCUSSION

The present study indicates that intraperitoneal injection of chondroitin sulphate accelerates the growth of tumour to some extent, but that the effect of hypodermal injection of the same agent is indistinct, and also that the concentration of chondroitin sulphate in the urine of intraperitoneally injected mice is higher than that of subcutaneously injected ones.

Kamei (1964) also reported that intraperitoneal injection of chondroitin sulphate promoted the growth of solid Yoshida ascites tumour in rats.

Ozzello *et al.* (1960) noticed the growth-promoting activity of acid mucopolysaccharides *in vitro* on a strain of human mammary carcinoma cells, their growth being in parallel with the rise in the concentration of acid mucopolysaccharides in the culture medium. The present author (Takeuchi 1966*a*, 1966*b*) reported that tumour growth in mice was accelerated in accordance with the increase in the concentration or amount of chondroitin sulphate solution which was injected prior to tumour inoculation into the same site.

A greater tumour-growth promoting effect of intraperitoneal injection of chondroitin sulphate as compared with the subcutaneous injection may be related to the chondroitin sulphate level in the serum of the mouse which, inferring from the concentration of the excreted chondroitin sulphate in the urine, is much higher in the intraperitoneal group than in the subcutaneous group.

The exact mechanism of the action of chondroitin sulphate on the growth of tumour is beyond the scope of the present study. However, the data obtained indicated that higher concentrations of acid mucopolysaccharides in the serum of mice is favourable to the growth of cancer cells.

SUMMARY

Using solid Ehrlich ascites tumour developed subcutaneously in SM mice, the effect of continuous administration of chondroitin sulphate on the growth of tumour and the relation between tumour growth and urinary excretion of chondroitin sulphate were studied.

It was observed that intraperitoneal injection of 1 ml. of 2% chondroitin sulphate accelerates the growth of tumour to some extent, but that the effect of hypodermal injection of the same agent is rather ambiguous. It was also shown that the concentration of chondroitin sulphate in the urine of intraperitoneally chondroitin sulphate-injected mice was higher than that of subcutaneously injected mice.

There may be some relationship between a greater tumour growth promoting effect of intraperitoneal injection of chondroitin sulphate as compared with subcutaneous injection and the chondroitin sulphate level in serum of the mouse which, inferring from the concentration of the excreted chondroitin sulphate in urine, is much higher in the intraperitoneal group than in the subcutaneous group.

The author owes a debt of gratitude to Prof. H. Tauchi for his advice and encouragement, and also to Dr. M. Kodama, Aichi Cancer Center Research Institute, for fruitful discussions of the problems.

### REFERENCES

Bergsterman, H.—(1955) Z. *Krebsforsch.*, **60**, 644.
Deutsch, H. F.—(1957) *J. biol. Chem.*, **224**, 767.
Kamei, H.—(1964) *Nagoya J. med. Sci.*, **27**, 142.
Kaziwara, K.—(1954) *Cancer Res.*, **14**, 795.
Leitner, J. G., and Kerby, G. P.—(1954) *Stain Technol.*, **29**, 257.
Ozzello, L., Lasfargeus, E. Y. and Murray, M. R.—(1960) *Cancer Res.*, **20**, 600.
Rich, C., and Meyers, W. P. L.—(1959) *J. Lab. clin. Med.*, **54**, 223.
Takeuchi, J.—(1961) *Stain Technol.*, **36**, 159.—(1965) *Nature Lond.*, **207**, 537.—(1966*a*) *Cancer Res.*, **26**, 797.—(1966*b*) *Br. J. Cancer*, **20**, 847.
Takeuchi, J., Kano, S., and Tauchi, H.—(1965) *Br. J. Cancer*, **19**, 353.